# Relationship between Lung Function and Metabolic Syndrome

**DOI:** 10.1371/journal.pone.0108989

**Published:** 2014-10-09

**Authors:** Wei-Liang Chen, Chung-Ching Wang, Li-Wei Wu, Tung-Wei Kao, James Yi-Hsin Chan, Ying-Jen Chen, Ya-Hui Yang, Yaw-Wen Chang, Tao-Chun Peng

**Affiliations:** 1 Division of Family Medicine, Department of Family and Community Medicine, Tri-Service General Hospital and School of Medicine, National Defense Medical Center, Taipei, Taiwan; 2 Division of Geriatric Medicine, Department of Family and Community Medicine, Tri-Service General Hospital and School of Medicine, National Defense Medical Center, Taipei, Taiwan; 3 Graduate Institute of Medical Sciences, National Defense Medical Center, Taipei, Taiwan; 4 Department of Occupational Safety and Hygiene, Fooyin University, Kaohsiung, Taiwan; University of Leicester, United Kingdom

## Abstract

Although the link between impaired lung function and cardiovascular events and type 2 diabetes mellitus has been recognized, the association between impaired lung function and metabolic syndrome has not been comprehensively assessed in the United States (U.S.) population. The aim of our study was to explore the association between impaired lung function and metabolic syndrome in a nationally representative sample of men and women. This cross-sectional population-based study included 8602 participants aged 20–65 years in the Third National Health and Nutrition Examination Survey (NHANES III). We examined the relationship between the different features of metabolic syndrome and lung function, including forced vital capacity (FVC) and forced expiratory volume in 1 second (FEV1). After adjusting for potential confounders such as age, body mass index, inflammatory factors, medical condition, and smoking status, participants with more components of metabolic syndrome had lower predicted values of FVC and FEV1 (*p* for trend <0.001 for both). Impaired pulmonary function was also associated with individual components of metabolic syndrome, such as abdominal obesity, high blood pressure, high triglycerides, and low high density lipoprotein (HDL) cholesterol (*p*<0.05 for all parameters). These results from a nationally representative sample of US adults suggest that a greater number of features of metabolic syndrome is strongly associated with poorer FVC and FEV1. In clinical practice, more comprehensive management strategies to address subjects with metabolic syndrome and impaired lung function need to be developed and investigated.

## Introduction

Impaired pulmonary function, which includes measurements of forced expiratory volume in one second (FEV1) and forced vital capacity (FVC), is mainly present in individuals with chronic obstructive pulmonary disease (COPD) and asthma [1]. Previous studies have shown that reduced FEV1 is a strong predictor of coronary heart disease, COPD-related mortality, and cardiovascular mortality [2]–[4]. Reduced FVC is also a marker for increased mortality in asymptomatic adults [5] or individuals with metabolic syndrome [6]. Recent years have seen an increased focus on metabolic syndrome in the prediction of lung function impairment. Previous data from Asian [7], [8] and European [9], [10] patients demonstrated a substantial association between impaired pulmonary function and metabolic syndrome. However, the diagnostic criteria and clinical features of metabolic syndrome varied by race. Few studies have focused on the U.S. general population to investigate the association between lung capacity and metabolic syndrome. In addition, important risk factors [8], [11], [12], such as gender, smoking status, biomarkers of inflammation, and C-reactive protein (CRP), were not fully taken into account in previous studies. The objective of our study was to investigate the independent relationship between impaired lung function and metabolic syndrome using by the Third National Health and Nutrition Examination Survey (NHANES III) sample, which is a well-designed population-based study with a large sample size of US adults.

## Methods

### Study population

Executed during the period 1988–1994, NHANES III consisted of a representative sample of the noninstitutionalized civilian US population, which was selected by using a multistage, stratified, and cluster sampling design [13]. All participants were interviewed for demographic, health, and dietary information within 2 months of initial enrollment. After a detailed home-based interview, participants were invited to receive pertinent examination sessions during which blood specimens were collected. For participants who were unable to attend the examination for health reasons, a blood sample was obtained during the home interview. We limited our analysis to participants aged 20–65 years who attended the medical examination and included the 8602 eligible subjects (4053 men and 4549 women) with complete information. To minimize confounding effects, participants with a history of asthma, chronic bronchitis, and emphysema were excluded. The NHANES III study received National Center for Health Statistics (NCHS) Institutional Review Board approval, and informed consent was acquired from participants prior to starting the study.

### Ethics statement

The NHANES study protocol was approved by the NCHS Institutional Review Board (IRB). Because our analysis exclusively used de-identified data, it was exempt from IRB review.

### Pulmonary Function Data

Spirometry was conducted using procedures based on the 1987 American Thoracic Society (ATS) recommendations [14]. Values used in this analysis included FVC and FEV1. The units of measurement for FVC and FEV1 values were liters. Each eligible subject performed at least five FVC maneuvers to meet the ATS acceptability and reproducibility criteria. Forced exhaled volumes were measured using a dry rolling-seal spirometer. All of the data were stored on digital tape, allowing the recalculation of all parameters based on ATS acceptability and reproducibility criteria [15]. We also used prediction equations to determine predicted FEV1 and FVC values. Predicted FVC and FEV1 values vary with the characteristics (age, height, gender, and race/ethnicity) of the specific population. For the United States, the predicted FVC and FEV1 were calculated using the reference equations developed by Hankinson et al. [15]. The spirometry system has been independently tested and found to exceed ATS spirometry equipment recommendations [16].

### Assessment of co-variates

Serum uric acid was measured using the Hitachi 737 automated multichannel chemistry analyzer (Boehringer Mannheim Diagnostics, Indianapolis, IN, USA). Chemical analyses of total cholesterol, triglycerides and HDL-C cholesterol (Hitachi 704 Analyzer) were performed by the Lipoprotein Analytical Laboratory at Johns Hopkins University, Baltimore, Maryland. LDL cholesterol levels were calculated using the Friedewald formula [17]. Serum glucose was determined by using an enzymatic reaction (Cobas Mira assay). Serum total bilirubin was measured by using the Hitachi 737 automated autoanalyzer (Boehringer Mannheim Diagnostics, Indianapolis, IN, USA). C-reactive protein was measured using latex-enhanced nephelometry. All protocols followed standardized methods that had documented accuracy with respect to Centers for Disease Control and Prevention (CDC) reference methods [13].

The participants were interviewed to collect information on sex, age, race/ethnicity (including non-Hispanic white, non-Hispanic black, Mexican-American, and other), body measurements (including height, weight, and waist), blood pressure, and medical conditions (including self-reported physician-diagnosed heart disease, and stroke). Body mass index (BMI) was calculated by dividing the individual's weight in kilograms by the square of their height in meters. Waist circumference was measured by trained NHANES staff using standard protocols. A brief questionnaire was used to determine the patient's smoking status. Detailed specimen collection and processing instructions are presented in the NHANES Laboratory Procedures Manual and are available on the NHANES website [13].

### Definition of metabolic syndrome

The revised National Cholesterol Education Program's Adult Treatment Panel III (NCEP: ATP III) defined metabolic syndrome as the presence of three or more of the following characteristics: (1) abdominal obesity: waist circumference >102 cm in men and >88 cm in women; (2) hypertriglyceridemia: ≥150 mg/dL (≥1.69 mmol/L);(3) reduced HDL cholesterol: <40 mg/dL (<1.03 mmol/L) for men and <50 mg/dL (<1.29 mmol/L) for women; (4) elevated blood pressure: systolic blood pressure ≥130 mm Hg or diastolic blood pressure ≥85 mm Hg; and (5) elevated fasting glucose: ≥100 mg/dL (5.6 mmol/L) [18].

### Statistical Analysis

All statistical analyses were performed using SPSS (Version 18.0 for Windows, SPSS, Inc., Chicago, IL, USA). Due to the complex survey design used in NHANES III, traditional calculations of statistical analyses based on the assumption of a simple random sample provided incorrect variance estimates and are not appropriate [19]. The “Complex Samples” procedure was used to incorporate sample weights and adjust for clusters and strata of the complex sample design. Predicted values of FEV1 and FVC were compared by the number of metabolic components or each individual components of metabolic syndrome. Based on published articles, influential demographic factors, and clinical standpoints, an extended-model approach was used for covariate adjustments. We used 2 models with progressive degrees of adjustment. Model 1 was adjusted for age and race/ethnicity. Model 2 was further adjusted for BMI, C-reactive protein, serum total bilirubin, serum uric acid, smoking, heart disease, and stroke. The P-values for trend tests were assessed by treating the components of metabolic syndrome as a continuous variable from 1 to ≧4 to observe the associations across increasing components of metabolic syndrome and lung function impairment.

## Results

### Demographics

A total of 8,602 participants (4053 males and 4549 females) were included in the study. The characteristics of the eligible subjects stratified by gender and the presence of metabolic syndrome are summarized in Table 1. The overall prevalence of metabolic syndrome was 26.85%. In our study, participants with metabolic syndrome were likely to be older and non-Hispanic white compared with individuals without metabolic syndrome among both men and women. The BMI, CRP, serum uric acid, and serum total bilirubin concentration were statistically significantly higher among those with metabolic syndrome than among those without the syndrome (*p*<0.001 for all factors).

**Table 1:** Characteristics of participants with or without metabolic syndrome.

Variables	Men (n = 4053)			Women (n = 4549)		
	Non-Metabolic syndrome N = 3164	Metabolic syndrome N = 889	P-value	Non-Metabolic syndrome N = 3128	Metabolic syndrome N = 1421	P-value
Continuous variables, mean (SD)						
Age (years)	35.21(10.55)	41.91(10.77)	<0.001	34.56(10.36)	40.73(10.58)	<0.001
BMI (kg/m^2^)	25.44(3.91)	31.09(5.62)	<0.001	25.16(5.18)	32.47(6.63)	<0.001
SBP (mmHg)	118.91(12.91)	132.50(16.13)	<0.001	109.81(12.80)	125.60(20.45)	<0.001
DBP (mmHg)	73.69(12.14)	83.24(11.59)	<0.001	67.11(10.93)	75.65(13.03)	<0.001
Waist circumference (cm)	89.78(10.82)	106.17(13.56)	<0.001	83.88(12.67)	101.85(13.49)	<0.001
Serum triglycerides (mg/dL)	142.36(114.73)	179.09(140.87)	<0.001	113.54(87.10)	139.24(114.29)	<0.001
HDL-C (mg/dL)	49.40(14.36)	40.27(12.31)	<0.001	57.85(14.64)	46.73(12.85)	<0.001
Serum glucose (mg/dL)	93.29(22.98)	111.98(43.76)	<0.001	87.28(18.569)	109.10(50.21)	<0.001
C-reactive protein (mg/dL)	0.33(0.51)	0.44(0.73)	<0.001	0.41(0.59)	0.66(0.80)	<0.001
Serum uric acid (mg/dL)	5.93(1.20)	6.42(1.41)	<0.001	4.27(1.06)	4.89(1.23)	<0.001
Serum total bilirubin (mg/dL)	0.71(0.37)	0.67(0.42)	0.004	0.52 (0.31)	0.47(0.22)	<0.001
FEV1 (L)	3.92(0.70)	3.64(0.76)	<0.001	2.90(0.54)	2.63(0.55)	<0.001
FEV1 % predicted	0.98(0.13)	0.94(0.15)	<0.001	0.99(0.14)	0.96(0.15)	<0.001
FVC (L)	4.87(0.81)	4.61(0.89)	<0.001	3.51(0.62)	3.22(0.63)	<0.001
FVC % predicted	1.00(0.12)	0.96(0.13)	<0.001	1.00(0.13)	0.97(0.14)	<0.001
FEV1/FVC	0.99(0.08)	0.98(0.09)	0.588	0.99(0.08)	0.99(0.08)	0.079
Categorical variables, n (%)						
Non-Hispanic white	1045(33.02)	311(34.98)	0.025	1163(37.18)	379(26.67)	<0.001
Heart disease	32(1.01)	8(0.89)	0.854	15(0.48)	20(1.40)	0.003
Stroke	21(0.66)	9(1.01)	0.284	8(0.26)	14(0.99)	0.004
Smoke	488(15.42)	198(22.27)	<0.001	12(0.38)	9(0.63)	0.250

SD, standard deviation;BMI, body mass index; SBP, systolic blood pressure; DBP, diastolic blood pressure; HDL-C, high-density lipoprotein cholesterol; WBC, white blood cell; CRP, C-reactive protein; FEV1, forced expiratory volume in 1; FVC, forced vital capacity.

### Metabolic components and pulmonary function test

Metabolic components such as SBP, DBP, waist circumference, serum glucose, and serum triglycerides were statistically significantly increased and HDL-C was significantly lower among those with metabolic syndrome compared with those without the syndrome (all of the parameters, *p*<0.001). In addition, pulmonary function tests, such as FEV1, FEV1 % predicted, FVC, and FVC1 % predicted were significantly lower among those with metabolic syndrome compared with those without the syndrome (all of the parameters, *p*<0.001). For the FEV1/FVC ratio, no statistically significant difference was noted between those with and without metabolic syndrome for both men and women (*p* = 0.588 and *p* = 0.079, respectively).

### Metabolic components and FVC predicted percentages

Results from models examining the association between metabolic components and FVC percent predicted values stratified by gender are presented in Table 2. As shown, there was a strong linear decrease in FVC percent predicted as the number of components of metabolic syndrome increased. After additional adjustment, the β coefficients of FVC percent predicted (%) for those with 1, 2, 3, and ≥4 features of metabolic syndrome were 0.006, −0.004, −0.021, and −0.049 in men and −0.017, −0.024, −0.041, and −0.073 in women, respectively (*p* for trend <0.001). In both males and females, abdominal obesity, high blood pressure, high triglycerides, high glucose, and low HDL-C were significantly associated with lower FVC percent predicted in fully adjusted models (all of the parameters, *p*<0.05).

**Table 2:** Regression coefficients of components of metabolic syndrome for FVC percent predicted.

Variables	Men				Women			
	Model 1		Model 2		Model 1		Model 2	
	β (95% CI)	P-value	β (95% CI)	P-value	β (95% CI)	P-value	β (95% CI)	P-value
Presence of metabolic syndrome	−0.034(−0.044, −0.024)	<0.001	−0.027(−0.038, −0.016)	<0.001	−0.034(−0.043, −0.025)	<0.001	−0.028(−0.038, −0.017)	<0.001
Number of metabolic syndrome components								
1	0.008(−0.011, 0.027)	0.398	0.006(−0.012, 0.025)	0.522	−0.017(−0.048, 0.015)	0.297	−0.017(−0.048, 0.014)	0.281
2	−0.005(−0.023, 0.014)	0.632	−0.004(−0.023, 0.015)	0.676	−0.026(−0.057, 0.005)	0.104	−0.024(−0.055, 0.008)	0.137
3	−0.025(−0.045, −0.004)	0.017	−0.021(−0.041, −0.001)	0.048	−0.044(−0.076, −0.012)	0.007	−0.041(−0.073, −0.008)	0.013
≧4	−0.054(−0.078, −0.030)	<0.001	−0.049(−0.074, −0.024)	<0.001	−0.081(−0.114, −0.048)	<0.001	−0.073(−0.107, −0.039)	<0.001
P for trend	<0.001		<0.001		<0.001		<0.001	
Components of metabolic syndrome								
Abdominal obesity	−0.041(−0.050, −0.031)	<0.001	−0.043(−0.056, −0.029)	<0.001	−0.024(−0.033, −0.016)	<0.001	−0.019(−0.030, −0.007)	0.002
High blood pressure	−0.019(−0.028, −0.011)	<0.001	−0.014(−0.022, 0.005)	0.002	−0.030(−0.041, −0.019)	<0.001	−0.023(−0.033, −0.012)	<0.001
High triglycerides	−0.026(−0.034, −0.018)	<0.001	−0.021(−0.029, −0.012)	<0.001	−0.022(−0.032, −0.012)	<0.001	−0.013(−0.023, 0.003)	0.011
Low HDL-C	−0.025(−0.034, −0.017)	<0.001	−0.020(−0.028, −0.011)	<0.001	−0.021(−0.030, −0.013)	<0.001	−0.014(−0.023, −0.006)	0.001
High glucose	−0.017(−0.026, −0.007)	<0.001	−0.014(−0.023, −0.004)	0.004	−0.045(−0.056, −0.034)	<0.001	−0.036(−0.047, −0.024)	<0.001

Model 1  =  age, race/ethnicity.

Model 2  = Model 1 + (body mass index, C-reactive protein, serum total bilirubin, serum uric acid, smoking status, heart disease, and stroke).

### Metabolic components and FEV1 predicted percentages

Results from models examining the association between metabolic components and FEV1 percent predicted stratified by gender are presented in Table 3. There was a significant inverse relationship between the number of components present and pulmonary function. After additional adjustment, the β coefficients of FEV1 percent predicted for those with 1, 2, 3, and ≥4 features of metabolic syndrome were 0.001, −0.011,−0.027, and −0.045 in men and 0.007, −0.007, −0.018 and −0.040 in women, respectively (*p* for trend <0.001). In both males and females, abdominal obesity, high blood pressure, high triglycerides, and low HDL-C were significantly associated with lower FEV1 percent predicted in fully adjusted models (all of the parameters, *p*<0.05). High glucose was significantly associated with lower FEV1 percent predicted in fully adjusted models in females.

**Table 3:** Regression coefficients of components of metabolic syndrome for FEV1 percent predicted.

Variables	Men				Women			
	Model 1		Model 2		Model 1		Model 2	
	β (95% CI)	P-value	β (95% CI)	P-value	β (95% CI)	P-value	β (95% CI)	P-value
Presence of metabolic syndrome	−0.029(−0.039, −0.018)	<0.001	−0.025(−0.036, −0.013)	<0.001	−0.027(−0.036, −0.017)	<0.001	−0.021(−0.032, −0.011)	<0.001
Number of metabolic syndrome components								
1	0.003 (−0.017, 0.023)	0.798	0.001(−0.020, 0.020)	0.990	0.008(−0.025, 0.040)	0.638	0.007(−0.026, 0.039)	0.678
2	−0.010 (−0.030, 0.010)	0.321	−0.011(−0.031, 0.009)	0.293	−0.008(−0.040, 0.025)	0.639	−0.007(−0.039, 0.026)	0.689
3	−0.028(−0.049, −0.006)	0.013	−0.027(−0.049, −0.004)	0.019	−0.020(−0.053, 0.013)	0.242	−0.018(−0.052, 0.015)	0.290
≧4	−0.045(−0.071, −0.020)	0.001	−0.045(−0.071, −0.018)	0.001	−0.045(−0.080, −0.011)	0.010	−0.040(−0.075, −0.004)	0.028
P for trend	<0.001		<0.001		<0.001		<0.001	
Components of metabolic syndrome								
Abdominal obesity	−0.035(−0.045, −0.025)	<0.001	−0.042(−0.056, −0.028)	<0.001	−0.023(−0.032, −0.014)	<0.001	−0.022(−0.034, −0.010)	<0.001
High blood pressure	−0.017(−0.026, 0.008)	<0.001	−0.012(−0.021, 0.003)	0.011	−0.028(−0.039, −0.017)	<0.001	−0.021(−0.033, −0.010)	<0.001
High triglycerides	−0.020(−0.029, −0.011)	<0.001	−0.016(−0.026, −0.007)	0.001	−0.026(−0.037, −0.016)	<0.001	−0.019(−0.030, −0.008)	<0.001
Low HDL-C	−0.025(−0.034, −0.016)	<0.001	−0.021(−0.031, −0.012)	<0.001	−0.022(−0.030, −0.013)	<0.001	−0.016(−0.025, −0.007)	<0.001
High glucose	−0.009(−0.019, 0.001)	0.061	−0.007(−0.017, 0.003)	0.146	−0.031(−0.043, −0.020)	<0.001	−0.022(−0.034, −0.010)	<0.001

Model 1 =  age, race/ethnicity.

Model 2  =  Model 1 + (body mass index, serum C-reactive protein, serum total bilirubin, serum uric acid, serum, smoking status, congestive heart failure, and stroke).

## Discussion

This study used a nationally representative sample of the U.S. population to examine the relationship between pulmonary function and metabolic syndrome, defined using revised ATP III criteria. Our findings indicated that both FEV1 and FVC were lower in proportion to the number of metabolic syndrome components the patients had. Notably, individual components of abdominal obesity, low HDL-C, high triglycerides, and high blood pressure were significantly associated with decreasing FVC and FEV1 in both males and females.

Metabolic syndrome is a complex disorder featuring chronic inflammation characterized by the constellation of abdominal obesity, hyperglycemia, hypertension, and dyslipidemia. Its definition varies by organization and expert group. The definition of the revised NCEP: ATP III is one of the most widely used. Although previous studies examined the association between individual components of metabolic syndrome and pulmonary function, the contribution of each component of metabolic syndrome to complications and comorbidity seems to differ between each race group. For instance, insulin resistance is related to blood pressure in white but not in black Americans [20]. Therefore, each component of metabolic syndrome may have a different influence on pulmonary function across racial groups. In an Italian study, HDL-C was the main predictor for pulmonary function impairment [9]. In a Japanese study, this relationship between lung function impairment and metabolic syndrome was thought to be due mainly to abdominal obesity and hyperglycemia in males [7]. In a Korean study, abdominal obesity, blood pressure, HDL-C, and fasting plasma glucose strongly influenced pulmonary function [8]. In an Australian study, poorer pulmonary function was also noted as metabolic syndrome components accumulated [21]. Our study, which was conducted in a U.S. population, revealed that metabolic syndrome was associated with impaired pulmonary function and that abdominal obesity, high blood pressure, high triglycerides and low HDL-C were significantly associated with decreasing FVC and FEV1 in both males and females.

Our finding that increased abdominal obesity was significantly associated with lung function impairment in both men and women was consistent with previous studies [12], [22]–[24]. Abdominal obesity is considered the core of the pathophysiology of metabolic syndrome [25]. The available data support a connection between metabolic syndrome and impaired pulmonary function, mainly via abdominal obesity. However, the identification of the definitive pathway and the exact pathophysiological mechanism to explain this association requires further evaluation. One potential explanation is that increased abdominal obesity directly affects thoracic and diaphragm compliance, which impairs lung function [26]. However, in a previous Japanese study, waist circumference was only significantly associated with impaired lung function in men [27]. Moreover, in Korea, sex differences in the association between waist circumference and pulmonary function were also recognized. Given that various distributions of abdominal and visceral fat accumulation were noted in different race populations and genders [25], our viewpoint of the varying weight of each component in different racial groups was confirmed. Another possible explanation for impaired pulmonary function mediated by abdominal obesity is that visceral fat may be a more specific marker [28]. Increased visceral fat was identified as the main factor that increases CRP concentrations [29]. In addition, increased serum CRP was positively correlated with each metabolic component and strongest with abdominal obesity [11], [30]. Serum CRP is also associated with impaired pulmonary function [31]. However, after considering CRP levels in our study, the relationship between impaired lung function and abdominal obesity was still evident.

Another important component, low HDL-C, was correlated with impaired pulmonary function in our study. In agreement with this observation, a study with 237 patients found that serum HDL-C had an inverse association with lower FEV1 and FVC [9]. The pathophysiology underlying this association remains unclear. Lower HDL-C levels are associated with the development of coronary heart disease because of the function of HDL-C in reverse cholesterol transport and anti-inflammation. It is tempting to speculate that the serum HDL-C level serves as a predictor for the decline of lung function, mainly due to its pleiotropic properties, including antioxidative function, inhibition of cytokine-induced expression of endothelial cell adhesion molecules, and suppression of the chemotactic activity of monocytes and lymphocytes [32], [33]. Klisic et al. also reported that significantly higher hs-CRP levels correlated with lower HDL-C levels [34], [35]. This observation implied that inflammation might be an early event in the decline of pulmonary function in individuals with low HDL-C.

The current analysis has a few limitations. First, this study was a cross-sectional survey that measured lung functions and metabolic components at a single time rather than recording long-term repeated observations. Although previous reports and biological plausibility consistently suggest that metabolic syndrome is associated with impaired lung capacity, a cross-sectional study design tends to leave uncertainty regarding the temporal sequence of exposure–outcome relations. Thus, confirming the relationship between the two prospective longitudinal data (the relationship between prior metabolic syndrome and incident impaired pulmonary function) would be valuable. Second, our samples were collected from 1988–1994. These samples may not accurately reflect today's U.S. population. However, based on a recent study of the prevalence of metabolic syndrome in the U.S. [36], individuals with metabolic syndrome were still likely to be older and non-Hispanic whites, which is similar to our sample. In addition, the current most useful FEV1 percent predicted and FEV percent predicted values were also calculated using the NHANES III reference equations developed by Hankinson et al. [15], which used a population similar to the one used in our study. Therefore, our sample result is applicable to today's U.S. population.

## Conclusions

Our findings highlight the notion that in the U.S., FVC and FEV1 are inversely associated with the accumulation of metabolic syndrome components and also independently associated with each component of metabolic syndrome. Therefore, this relationship might receive more attention and even urge action to be taken on metabolic components in the context of poor pulmonary function. The results of this study warrant the development and investigation of comprehensive management strategies.
